# Genetic analysis of the *Trichuris muris*-induced model of colitis reveals QTL overlap and a novel gene cluster for establishing colonic inflammation

**DOI:** 10.1186/1471-2164-14-127

**Published:** 2013-02-26

**Authors:** Scott E Levison, Paul Fisher, Jenny Hankinson, Leo Zeef, Steve Eyre, William E Ollier, John T McLaughlin, Andy Brass, Richard K Grencis, Joanne L Pennock

**Affiliations:** 1 Gastrointestinal Sciences, Institute of Inflammation and Repair, Faculty of Medicine and Human Sciences, University of Manchester, 4.004 AV Hill Building, Oxford Road, Manchester M13 9PT, UK; 2 Bioinformatics Scientist, Oncology, AstraZeneca, Cheshire, UK; 3 Centre for Integrated Genomic Medical Research, Institute of Population Health, Faculty of Medicine and Human Sciences, University of Manchester, Manchester, UK; 4 Bioinformatics, Faculty of Life Sciences, University of Manchester, Manchester, UK; 5 Arthritis Research UK Epidemiology Unit, Institute of Inflammation and Repair, Faculty of Medicine and Human Sciences, University of Manchester, Manchester, UK; 6 School of Computer Sciences, University of Manchester, Manchester, UK; 7 Faculty of Life Sciences, University of Manchester, Manchester, UK

**Keywords:** Trichuris muris, Colitis, Genetic susceptibility, Cdcs1, Crohn’s

## Abstract

**Background:**

Genetic susceptibility to colonic inflammation is poorly defined at the gene level. Although Genome Wide Association studies (GWAS) have identified loci in the human genome which confer susceptibility to Inflammatory Bowel Disease (Crohn’s and Ulcerative Colitis), it is not clear if precise loci exist which confer susceptibility to inflammation at specific locations within the gut e.g. small versus large intestine. Susceptibility loci for colitis in particular have been defined in the mouse, although specific candidate genes have not been identified to date. We have previously shown that infection with *Trichuris muris* (*T. muris*) induces chronic colitis in susceptible mouse strains with clinical, histological, and immunological homology to human colonic Crohn’s disease. We performed an integrative analysis of colitis susceptibility, using an F2 inter-cross of resistant (BALB/c) and susceptible (AKR) mice following *T. muris* infection. Quantitative Trait Loci (QTL), polymorphic and expression data were analysed alongside *in silico* workflow analyses to discover novel candidate genes central to the development and biology of chronic colitis.

**Results:**

7 autosomal QTL regions were associated with the establishment of chronic colitis following infection. 144 QTL genes had parental strain SNPs and significant gene expression changes in chronic colitis (expression fold-change ≥ +/-1.4). The *T. muris* QTL on chromosome 3 (*Tm3*) mapped to published QTL in 3 unrelated experimental models of colitis and contained 33 significantly transcribed polymorphic genes. Phenotypic pathway analysis, text mining and time-course qPCR replication highlighted several potential *cis-*QTL candidate genes in colitis susceptibility, including *FcgR1*, *Ptpn22*, *RORc,* and *Vav3*.

**Conclusion:**

Genetic susceptibility to induced colonic mucosal inflammation in the mouse is conserved at *Tm3* and overlays *Cdcs1.1*. Genes central to the maintenance of intestinal homeostasis reside within this locus, implicating several candidates in susceptibility to colonic inflammation. Combined methodology incorporating genetic, transcriptional and pathway data allowed identification of biologically relevant candidate genes, with *Vav3* newly implicated as a colitis susceptibility gene of functional relevance.

## Background

Many diseases result from the complex interaction of environmental and genetic factors (e.g. Crohn’s disease, diabetes mellitus) [1,2]. Phenotypic expression is influenced by multiple genes, which individually may increase or decrease the probability of disease development. Gene variation and gene-gene interactions, additionally results in non-linear contributions to phenotypic variation. Discovering the genetic architecture of complex traits thus represents a true challenge [3] and requires collaborative multi-disciplinary investigation and a variety of experimental approaches [4,5]. The exploration of new animal models of colitis with well-defined phenotypes and homology to human pathology, provide a comparative approach to refine biological discoveries for subsequent human translation.

*Trichuris muris*, a natural intestinal parasite of mice has been extensively studied as a model for human whipworm (*Trichuris trichiura*) infection. In dissecting the immune response to *Trichuris* infection, a paradigm of resistance and susceptibility to chronic colonic inflammation has emerged [6]. Following the ingestion of parasite ova, acute colitis develops in all mice, but it is the genetic composition of mouse strain which dictates the presence of colitis. BALB/c mice mount immune-mediated T_H_2 dependent parasite expulsion (IL4, IL5 and IL13 expression) [6,7] with full resolution within 20 days. Conversely AKR mice sustain a chronic *Trichuris* infection, respond with a T_H_1 immune response (IFNγ, IL12), and subsequent establishment of colitis [8]. These polarized outcomes occur despite identical treatment and conditioning, and are almost certainly determined by host genetic variation. Importantly, we have recently characterised differences in colonic tissue transcription between susceptible and resistant mice and demonstrated phenotypic, immunological and biological pathway homology to human Crohn’s disease [9]. These data present *T. muris* infection not as an aetiological factor in the pathogenesis of Crohn’s disease, nor solely a model of infection but as a viable and relevant colitis model to investigate and study mucosal inflammation.

The multifactorial and complex nature of Crohn’s disease remains to be fully characterised, but it is evident that disease can be initiated anywhere along the digestive tract. It is likely that precise environmental triggers determine the site of initiation, but it is also possible that host genetics play a part. A variety of experimental models have been developed to study pathogenic mechanisms responsible for the induction and perpetuation of Crohn’s disease. Phenotypic and biological factors common between colonic Crohn’s disease and chronic *T. muris* induced colitis, present a novel opportunity to characterise the genetic architecture central to disease susceptibility in the colon. The aim of the current study was to identify genome wide genetic elements and mechanistic pathways which underpin the development and maintenance of such chronic inflammation.

## Results

### Systemic and colonic phenotyping of chronic *T. muris* colitis in an F2 population of resistant and susceptible mice

**Figure 1:**
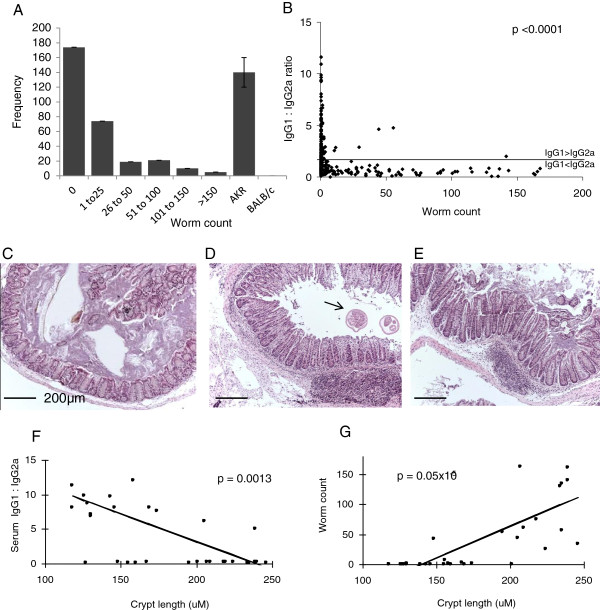
**Phenotype data. A)** Colonic worm burden across the F2 population with parental strains. **B**) Serum IgG1:IgG2a ratio was significantly different between resistant (0 worms) and susceptible (>0 worms) groups. **C**) Normal colonic histology in resistant mouse with predominant serum IgG1 (x100 magnification. H&E stain. Bar = 200μm). Histological mucosal and submucosal colonic inflammation, with crypt hyperplasia and elongation, seen in the vicinity of (**D**), and away from (**E**) *T. muris* colonic worms (arrow). **F**) Serum IgG1:IgG2a ratio, and **G**) colonic worm count correlate with histological inflammatory features.

An F2 inter-cross of resistant (BALB/c) and susceptible (AKR) mice was phenotyped 35 days post-infection, a time-point when chronic inflammation is established in susceptible mice (Figure 1). Colonic worm burden was not normally distributed; the majority of animals were resistant, with the largest worm burdens harboured by a small number of individuals (Figure 1A). This pattern of worm load distribution is indicative of an out-bred cohort [10]. Serum parasite-specific IgG1 (T_H_2 specific) and IgG2a (T_H_1 specific) was measured. Many individuals had a combination of both serotypes. To determine the predominant phenotype expressed a serum IgG1:2a ratio was calculated. A highly significant difference was observed between the mean of IgG1:2a ratio in resistant and susceptible mice (Mann Whitney U test, p < 0.0001, Figure 1B). At day 35 post-infection, 83% (110/133) of individuals with persistent worm burden demonstrated serum antibody titre IgG1 < IgG2a, indicative of a polarised T_H_1 immune response. A dominant T_H_2 immune response (IgG1 > IgG2a) correlated with worm expulsion. Furthermore, females demonstrated more resistance compared to males; females had significantly fewer worms at D35 post infection (Additional file 1: Figure S1A) and significantly higher IgG1:2a ratio (p < 0.0001, Additional file 1: Figure S1B) indicative of a dominant Th2 immune response.

Colonic histological assessment demonstrated persistent *T. muris* infection and large bowel inflammation. Mild-to-moderate inflammatory changes included: transmural tissue oedema and associated leukocytic infiltration (lymphocytes, macrophages, neutrophils); prominent mucosal and submucosal reactive lymphoid aggregates; colonic crypt hyperplasia and hypertrophy (Figure 1C-E). Significant correlation between histological parameters of inflammation (e.g. crypt length), immune response phenotype (Figure 1F: Spearman’s Rs = -0.54) and worm burden (Figure 1G: Spearman’s Rs = 0.84), were demonstrated. 98.5% of mice with persistent helminthosis demonstrated colonic inflammatory changes.

### Whole genome Linkage analysis

In total 7 QTL demonstrated significant correlation between susceptibility phenotype and genotype. Chromosomal locus, LOD scoring, trait correlation and the number of genes found within each QTL were defined (Table 1). The majority of QTL were associated with both a T_H_1 pro-inflammatory immune response, as reflected in low IgG1:IgG2a ratio, and persistent worm burden. *Tm10* however, was solely indicative of continued worm persistence. *Tm17* demonstrated the most significant LOD score, overlying the major histocompatibility complex (MHC).

**Table 1:** **Summary of *Trichuris muris *QTL (*Tm*) found across the genome**

**T. Muris QTL**	**Murine chrom**	**Left marker**	**Right marker**	**LOD**^**a**^	**Trait**	**Dominance**	**Gene no. within QTL (Ensembl)**	**AKR vs BALB/c genes with SNPs**
Tm1	1	*D1Mit33*	*D1Mit36*	2.39	Serum IgG’s Worm count	−8.3	90	68
Tm3	3	*D3Mit156 92.4Mbp*	*D3Mit79 118.3Mbp*	2.80	Serum IgG’s Worm count	8.4	342	270
Tm4	4	*D4Mit166*	*D4Mit12*	3.23	Serum IgG’s	−5.3	311	289
Tm10	10	*D10Mit14*	*D10Mit35*	4.10	Worm count	-	27	24
Tm11	11	*D11Mit99*	*D11Mit61*	4.14	Serum IgG’s Worm count	−0.3	265	182
Tm12	12	*D12Mit285*	*D12Mit201*	4.7	Serum IgG’s Worm count	−10.7	95	75
Tm17	17	*D17Mit175*	*D17Mit176*	8.04	Serum IgG’s Worm count	−5.7	287	251

^a^For non-parametric traits, Kruskal Wallis values were converted to LOD according to published methods [13].

**Figure 2:**
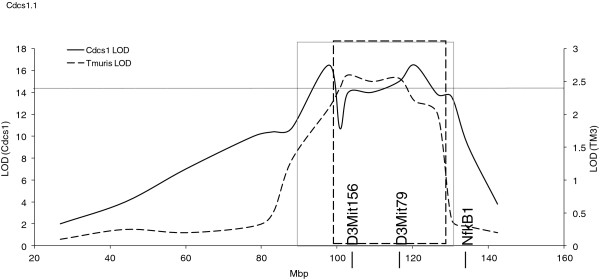
***Tm3 *overlays the colitic *Cdcs1 *QTL.** Previous congenic analysis defined *Cdcs1* between 87.1 and 131.1 Mbp (solid box), and overlays *gpdc1* (dashed box), a mouse QTL which also correlates with spontaneous colitis. *T. muris* QTL *Tm3* (broken line) lies between D3Mit156 (92 Mbp) and D3Mit79 (118 Mbp), outside the location of a previously defined candidate gene NfkB1 (135.1 Mbp). The threshold for suggestive correlation is shown for *Tm3* at LOD 2.4.

Of particular interest, *Tm3* (92.4-118.3 Mbp, chromosome 3) demonstrated complete overlap with a susceptibility locus identified in three unrelated murine models of spontaneous experimental colitis: G-protein alpha inhibitory 2 chain knock-out (*Gnai2*^-/-^) mice (*Gpdc1* locus) [11]; C3H/HeJBir IL10-deficient mice (*Cdcs1* locus) [12]; T-bet^-/-^Rag2^-/-^ double-deficient mice that resemble ulcerative colitis (TRUC) (*Cdcs1* locus) [13]. The *Cdcs1* region has been shown to contain at least three distinct regions [14,15]. Here, we show complete overlap of *Tm3* with *Cdcs1.1* (Figure 2), a region shown to contribute strongly to the severity of colitis in C3H/HeJBir mice [14,15].

### Prioritization of QTL candidate genes via pathway-driven workflow analysis

**Figure 3:**
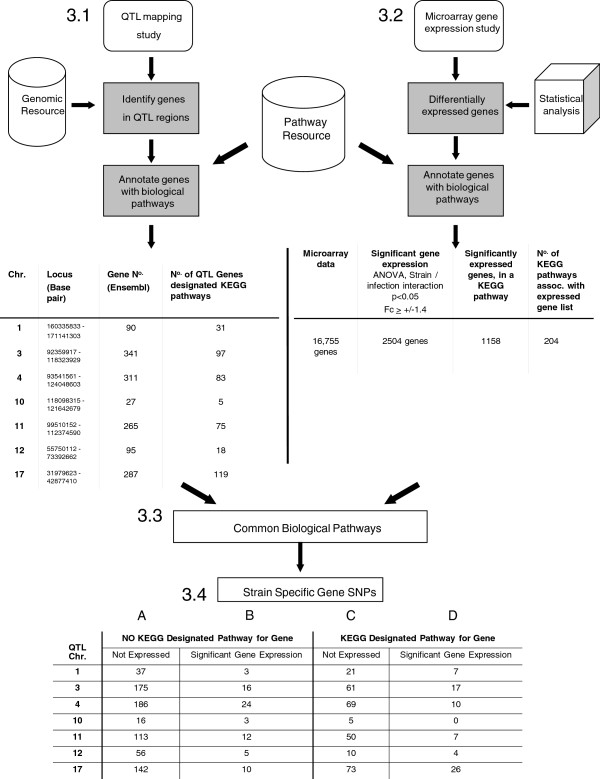
**Flow diagram showing unbiased identification of candidate genes in identified QTL. 3.1:** Genes within QTL were identified and assigned biological pathways. **3.2:** In parallel, genes in QTL with different relative expression between parental strains were assigned biological pathways. **3.3** &**3.4:** Genes within commonly identified pathways were ranked according to SNP number (AKR vs BALB/c http://www.sanger.ac.uk).

Schematic representation and key stage data from each of the *qtl_to_pathway*[16] and *refseq_ids_to_pathways*[17] workflows are shown (Figure 3). In total, 1419 genes were identified within the 7 *T. muris* QTL. Genes attributed to one or more KEGG biological pathways were determined (Figure 3.1). Simultaneously, of 5476 genes with significant transcriptional differences during *T. muris* infection [9], 2504 genes displayed either an up-regulated or down-regulated change in expression of ≥ 1.4 fold (i.e. a 40% or greater change in expression over naïve controls, Figure 3.2). Biological KEGG pathways associated with significant gene expression data were determined. In total, 1158 of these genes (46%) were involved in 204 separate KEGG pathways (Figure 3.3).

The cross correlation of functional pathways containing QTL genes and genes demonstrating significant expression were identified, linking genotype and phenotype trait interactions (Figure 3.3). Finally, polymorphic genes between parental AKR and BALB/c mice were identified within each locus (Figure 3.4).

As an example, 344 Ensembl gene ID’s were detected within *Tm3*. Of these, 97 (28%) were designated as functionally important within molecular interaction networks, as assigned by the KEGG pathway database. Significantly expressed microarray genes were similarly assigned biological pathways. For *Tm3*, the cross correlation of common pathway data and the exclusion of any gene which lacked SNPs between parental strains, identified 17 Quantitative Trait genes (Figure 3.4, Column D). In comparison, 61 KEGG-assigned polymorphic genes did not demonstrate any change in transcriptional activity (Column C). Of the genes yet to be allocated a KEGG pathway, 16 of 191 genes displayed significant transcription (Column B). The same process was undertaken for all 7 QTL (Figure 3).

### Chromosome 3 candidates

Analysis of *Tm3*, revealed 33 polymorphic genes with significant transcriptional changes during infection. Candidate genes were analysed in two distinct subsets; those with and those without a designated KEGG pathway. Of the 33 genes, 17 demonstrated a central mechanistic role within one or more KEGG pathway (Table 2). *Vav3* was associated with the highest number of pathways (n = 7). Candidate genes were ranked according to the number of SNPs that occurred between AKR and BALB/c strains. *Vav3* was the gene with the highest number of SNP variants (n = 2047).

**Table 2:** **Significantly expressed *Tm3 *genes possessing strain-specific SNPs and a designated biological (KEGG) pathway**

**Gene**	**KEGG designated pathway**	**SNP no.**	**Gene**	**KEGG designated pathway**	**SNP no.**
**Vav3**	Chemokine signalling pathway	2047	**Fcgr1**	Fc gamma R-mediated phagocytosis	18
	Fc gamma R-mediated phagocytosis			Hematopoietic cell lineage	
	Focal adhesion			Leishmaniasis	
	Leukocyte transendothelial migration			Phagosome	
	T cell receptor signalling pathway			Systemic lupus erythematosus	
	B cell receptor signalling pathway		**Prpf38b**	Spliceosome	9
	Regulation of actin cytoskeleton				
**Hmgcs2**	Butanoate metabolism	134	**Vcam1**	Cell adhesion moleculaes (CAMs)	8
	Metabolic pathways			Leukocyte transendothelial migration	
	Synthesis and degradation of ketone			Malaria	
	Terpenoid backbone biosynthesis		**Cdc14a**	Cell cycle	7
	Valine, leuckine and isoleucine degradation				
**Ctss**	Antigen processing and presentation	120	**Dbt**	Metabolic pathways	2
	Lysosome			Valine, leucine and isoleucine degradation	
	Phagosome				
**Ap4b1**	Lysosome	92	**Gstm7**	Drug metabolism – cytochrome P450	2
**Rorc**	Circadian rhythm	76		Glutathione metabolism	
				Metabolism of xenobiotics by cytochrome p450	
**Gstm3**	Drug metabolism – cytochrome P450	73	**Hsd3b6**	Metabolic pathways	2
	Glutathione metabolism			Steroid hormone biosynthesis	
	Metabolism of xenobiotcis by cytochrome P450				
**Dpyd**	Beta-Alanin metabolism	24	**Fmo5**	Drug metabolism – cytochrome p450	1
	Drug metabolism – other enzymes				
	Metabolic pathways		**Gstm6**	Drug metabolism – cytochrome p450	1
	Pantothenate and CoA biosynthesis				
	Pyrimidine metabolism		**Hist2h2be**	Systemic lupus erythematosus	1

The 16 genes with no KEGG pathway association were subsequently analysed using a workflow-based text-mining approach, to allow prioritization according to known biological roles in inflammation or gut immunology. Genes were ranked according to a cosine vector score (see Methods), estimating the significance of correlation between candidate gene and phenotype. SNP variation between parental strains was also considered. As a result, additional proposed candidates included: *Ptpn22*, *S100a10*, and *Slc22a15* (Table 3).

**Table 3:** Significantly expressed genes possessing strain-specific SNPs but as of yet, undesignated a biological (KEGG) pathway

**Gene**	**Text mining (cosine vector score)**	**SNP no.**
**S100a10**	**0.248**	**62**
Slc22a150	0.0219	19
Selenbp1	0.0065	19
Mov10	0.0063	3
Ppm1j	0.0013	3
Pogz	0.0009	8
Extl2	0.0008	2
Igsf3	0.0007	177
Ptpn22	0.0006	276
Selenbp2	0.0004	21
Wdr	0.0004	1
Cd53	0.0002	92
Cttnbp2nl	0	55
Golph3l	0	34
4933421E11Rsk	0	19
Eps8l3	0	1

Genes ranked according to text-mining significance, and SNP number for each gene noted. (QTL Chromosome 3, TM3). The top candidate is highlighted in bold: S100a10 had the highest cosine vector score demonstrating maximum relevance in the literature.

Transcript profiling: Microarray data has been submitted to ArrayExpress. Accession number E-MEXP-3098.

### Candidate gene validation

Quantitative PCR analysis was undertaken independently in infected parental strains (days 0, 7, 14, 21, and 35 post-infection) to validate microarray data (Tables 2 and 3).

**Figure 4:**
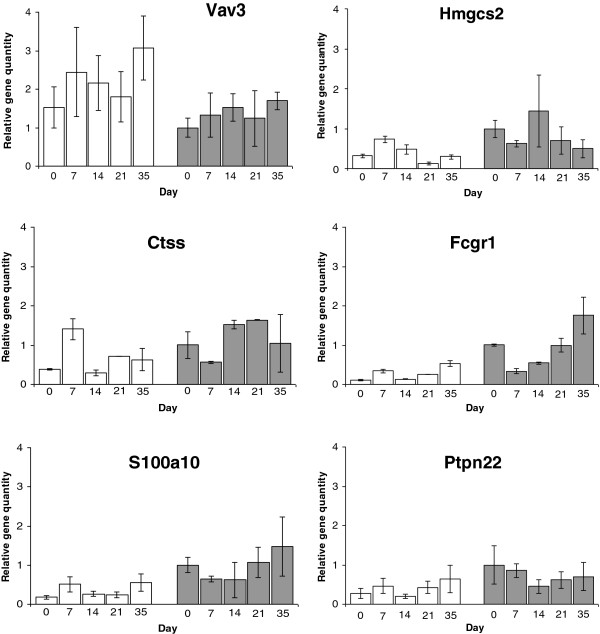
**Colonic *Tm3 *gene expression by independent qPCR.** Results are displayed relative to naïve resistant BALB/c, following standardization and normalization of samples against housekeeper gene (β-actin). Shown are the top 3 candidates by pathway & SNP analysis (Table 1: *Vav3, Hmgcs2 & CTSS*), the current strongest candidate gene from the literature (*Fcgr1*), the candidate with the highest text mining score but no designated biological pathway (Table 3: *S100a10*) and the candidate with the most SNPs but as yet without a designated pathway (Table 3: *Ptpn22*). Open bars denote susceptible AKR, shaded bars denote resistant BALB/c.

For gene candidates, *Vav3, Ptpn22*, *FcgR1* and *S100a10,* qPCR corroborated up-regulated expression found in susceptible AKR on day 35 post infection (Figure 4). Likewise, the down-regulation of *Hmgcs2* was confirmed by qPCR. Microarray analysis of *Ctss* and *RORc* demonstrated down regulation of colonic gene expression in chronically affected individuals, which could not be replicated (data not shown for *RORc*).

## Discussion

*Trichuris muris*-induced colitis represents a tractable murine model for understanding the patho-biological mechanisms of chronic intestinal inflammation [9,19]. The use of Quantitative Trait Loci (QTL) mapping based on continuous phenotypic variation has proved a useful technique in many murine polygenic traits including intestinal inflammation [12,20,21]. Yet, of more than 2000 QTL documented within the mouse genome database [22] fewer than 1% of studies have actually been characterized at a gene or molecular level, due to the small effect size of the susceptibility locus in question (<10% penetrance), or the large interval size defined [22]. New multi-factorial approaches have been discussed in the literature [23] and demonstrate that understanding complex genetic traits requires an integrative analysis.

Specific steps were taken in our experimental design to consider recent reports concerning the QTL/microarray approach in the identification of QTL candidate genes [23]. First, QTL were defined with regards to experimental phenotype (pQTL), and correlated with transcriptional expression activity in parental strains. Second, the use of high density Affymetrix exon array, which targets approximately 40 exonic probes per gene, overcame any problem of potential allelic-biased probe binding. Third, a hypothesis-free pathway analysis, backed up by additional text-mining, was employed in the secondary filtering of potential candidate genes to reduce bias. Fourth, any genes lacking polymorphisms (coding and non-coding) between parental strains were excluded from analyses, and lastly, positional overlap with a previously replicated major colitis susceptibility quantitative trait locus (*Cdcs1*) prioritised *Tm3* for targeted analysis.

With regards to this shared locus, *Cdcs1* on chromosome 3 was first noted in a QTL study of spontaneous colitis using IL10 deficient mice [12]. This locus has been shown to contain at least 3 distinct regions (*Cdcs1.1*, *1.2* &*1.3*) that contribute to a severe colitic phenotype [14,15]. Interestingly, all three regions contribute to caecal and proximal colonic inflammation strongly suggesting that this locus is a colitis ‘hotspot’ for susceptibility and/or regulation. Here we show complete overlap of *Tm3* with *Cdcs1.1* (Figure 2). Although *NF-kB1* has been suggested as a candidate gene for the *Cdcs1* locus, it is clear that it is not responsible entirely for the severe pathology observed [15]. To date, *FcgR1* remains the key candidate gene described in the *Cdcs1.1* region [14,15] and is corroborated by our findings. Additional association with colitis susceptibility in Gnai2^-/-^ mice [11] suggests that this locus may govern key inflammatory pathways in disease development, irrespective of trigger. QTL mapping specifically highlighted the *Cdcs1.3* region in the spontaneous colitis and colorectal cancer development of TRUC mice [13]. However, more distal colonic disease (distal third of the colon) or the potential for malignant transformation may not be represented at this sub-locus.

We have shown that at least 6 biologically significant and polymorphic candidate genes lie within the *Cdcs1.1* autosomal region. Importantly, 4 of these candidate genes are key in pathways relevant in the context of human Crohn’s disease (*FcgR1*, *Vav3*, *Vcam1* and *Ctss*), a disease with highly similar pathology to both the IL10 deficient and *T. muris* models of colonic inflammation [9,24]. The remaining 2 genes are highly polymorphic and known to be important in inflammation (*RORc*, *Ptpn22*). As individual candidate genes, each demonstrates interesting biological functionality that could play a role in mucosal inflammation. For instance, *FcgR1* codes for a high affinity IgG receptor, key to IgG2a-induced phagocytosis and antigen specific immune responses. In the mouse, *FcgR1* has been associated with autoimmune disorders such as rheumatoid arthritis and bacterial infection [25]. In humans the closely related *FcgR2a* and *FcgR3* have been associated with IBD [26]. The protein tyrosine phosphatase gene (*Ptpn22*) is of particular interest, as in humans a mis-sense SNP (C1858T) has already demonstrated strong correlation with rheumatoid arthritis [27], type-1 diabetes mellitus [28], and other autoimmune disease [29]. Interestingly, the C1858T gene variant is not associated with the establishment of human Crohn’s disease [30] and may even represent protection [31]. In our study, *Ptpn22* demonstrated progressively increased expression within the colonic tissue of susceptible mice following the establishment of colitis.

The unbiased approach we have used to select candidate genes has also highlighted a gene whose currently assigned pathway (circadian rhythm) does not overtly relate to mucosal inflammation. The Retinoic acid-related orphan receptor-C (*RORc*/*RORγ*) gene encodes for RORγt (RORγ2), a lineage-specific transcription factor of CD4+ T_H_17 cell differentiation [32]. Excessive T_H_17 cell activity has been implicated in both autoimmune [33] and inflammatory bowel diseases [34].

Finally, *Vav3* was the primary candidate revealed by integrative pathway and SNP analysis and is of particular interest, as in six week old *Vav1/2/3* triple knockout mice altered gut enterocyte differentiation and morphology has been shown, along with spontaneous colitis and ulceration in the caecum and ascending colon [35]. *Vav3* is also involved in at least 7 known biological pathways, all of which could play a role in mucosal homeostasis and regulation. Some of these pathways involve other candidate genes in this region, for instance *FcgR1* (Fc-gamma receptor mediated phagocytosis), *Vcam1* (leukocyte transendothelial migration, focal adhesion) and *Ptpn22* (negative regulation of T-cell receptor signalling) [36,37]. We hypothesise therefore that *Cdcs1* is in fact a ‘colitis hotspot’ containing several genes which if dysregulated through genetic variation, could adversely affect gut inflammation. It is possible that the specific candidate genes for each colitis model are not the same. However, the biological interaction between genes at this locus, demonstrates the importance of *Cdcs1* and why this region appears in unrelated models of gut inflammation.

Interestingly, *Tm3* (*Cdcs1*) does not correlate with any known nematode infection susceptibility QTL, but instead appears exclusive to colonic inflammatory disease.

For instance, expulsion and resistance to the small intestinal nematode *Heligmosomoides bakeri* in mice has been characterized at murine chromosome 1 and 17 [38] corresponding to *Tm1* and *Tm17*. Similarly, a study of *Trichinella spiralis* infection in rats, which causes acute and transient small bowel inflammation, identified a single significant QTL region homologous to the murine chromosome 1 locus (*Tm1*) [39]. Lastly, resistance to small bowel and abomasum/gastric nematode infections of sheep, have highlighted a number of suggestive QTL [40-42], homologous to *Tm17*, and downstream of *Tm10*. All studies demonstrated that resistance/susceptibility to GI nematode infection is under multi-genetic control, with MHC and non-MHC loci important in outcome [43]. However, these studies also highlight the importance of the *Cdcs1* locus with the establishment of a large bowel inflammatory phenotype, separate to precise anti-parasitic mechanisms.

In conclusion, we have corroborated three previously published studies which associate the locus *Cdcs1* with colonic mucosal inflammation in the mouse. Furthermore, we have shown that in the AKR and BALB/c, genetic variation in this region has the potential to affect mucosal homeostasis through several different pathways. Most importantly, we have demonstrated that an unbiased integrative analysis can be beneficial in candidate gene identification and prioritization, particularly *cis*-regulated genes, even in large regions. This approach is particularly useful for hypothesis generation, and has positionally implicated *Vav3* as a biologically relevant gene candidate in colitis.

## Methods

### Animals

Mice were housed with free access to food and water under specific pathogen free conditions. All experiments were performed under regulations of The UK Home Office Animals (Scientific Procedures) Act of 1986.

For QTL analysis, AKR/OlaHsd (susceptible, hereafter referred to AKR) and BALB/cOlaHsd (resistant, hereafter referred to BALB/c) mice (Harlan Olac, UK) were interbred. To generate an F1 population of mice, equal numbers of AKR males vs BALB/c females (F1a offspring), and AKR females vs BALB/c males (F1b offspring) were mated. At least 50 breeding-pairs of F1-mice were then interbred. All F1 vs F1 breeding was performed over the same time-period. To maintain genetic balance, F1a males were bred with F1a and F1b females; and, F1b males with F1a and F1b females. A single generation of 307 F2 mice (male and female) was created for study. All F2 mice were infected at the same time with *T. muris* ova at 6-8 weeks of age.

### Parasites

*Trichuris muris* parasites were harvested and ova collected and maintained as previously described [18]. All infected mice received 300 *T. muris* ova in distilled water (200 μl) by oral gavage.

### QTL phenotyping

Phenotypic analysis was performed for all 307 F2 mice. Day 35 post-infection, serum samples and intestines were taken at autopsy. Resistance (0 worm load) and susceptibility (>0 worms) were defined. All worm counts were performed by a single investigator over 1 week from caeca frozen at autopsy. This method of storage and counting is used routinely for large experiments and does not affect quantification. Parasite-specific antibody ELISA was performed as described previously [44], using in-house *T. muris* excretory-secretory (ES) protein. *T. muris* specific IgG1 (T_H_2 specific, driven by IL4) and IgG2a (T_H_1 specific, driven by IFNγ) optical density (OD) was measured simultaneously for all samples. All 307 serum ELISAs were performed in one run. For histology, 0.5cm of whole colonic segments from the proximal ascending colon was snap-frozen, thawed in 4% paraformaldehyde, paraffin embedded, and 5μm transverse tissue sections stained with Haematoxylin and Eosin (H&E) simultaneously. 50 randomly assigned colonic specimens were assessed. Proximal colonic specimens were scored according to colonic crypt length (μm), immune cell infiltration and tissue inflammation by a single investigator. Colonic crypt length per individual was taken as a mean across at least 20 crypt units and 3 separate sections. Crypt units were measured using Image-J software [45]. Spearman’s rank correlation coefficient was performed to measure the statistical dependence of (a) worm count and colonic crypt length variables, and (b) IgG1:IgG2a ratio and crypt length variables.

### DNA extraction

DNA was isolated (Promega Wizard DNA isolation kit) from tail snips digested in proteinase K digestion buffer (20 mg/ml). DNA concentration was determined by Nanodrop spectrophotometer and then stored at -80°C until analysis.

### Linkage Map

165 polymorphic murine microsatellite markers distinguishing between AKR and BALB/c were selected [46]. Whole genome coverage was 85% and median inter-marker distance 12.3 cM. Conversion of marker positions from recombination fraction (cM) to physical position (Mb) was achieved using the Ensembl database [47].

### Microsatellite amplification and genotype analysis

Forward polymerase chain reaction (PCR) primers were fluorescently labelled with 6-FAM, HEX or NED (MWG Biotec, Applied Biosystems). 25 ng of genomic DNA was used for each marker. Semi-automated analysis of genotypes on pooled panels of PCR products was performed using an Applied Biosystems 3100 Capillary sequencer with Genescan analysis and Genotyper software.

### QTL analysis

IgG1:IgG2a ratios were log_10_ transformed to achieve parametric distribution. Median, mean and kurtosis values were calculated using QStat, Windows QTL Cartographer 2.5. Normalised data were analysed using multiple interval mapping to optimize and refine QTL positions. A genome-wide permutation test (1000 repeats) determined thresholds for significance; a logarithm of odds (LOD) score of 4.0 or a p value of <5.2×10^-5^ was considered significant. A LOD score of 2.5 or p value of 1.6×10^-3^ was considered suggestive of linkage according to published guidelines [48]. All significant LOD scores were confirmed by 1-way ANOVA with pairwise comparison, using the Bonferroni correction method. Kruskal-Wallis analysis was used for worm burden and IgG2a data, and converted to an LOD score [49].

### Genome-wide colonic transcriptional activity of parental murine strains

Naïve and infected 6-to-8 week old male AKR and BALB/c mice (Harlan Olac, UK) were monitored through to day 35 post-infection (n = 6, 3 experimental replicates for each conditional cohort) as described previously [9]. 3 replicate pooled samples of colonic RNA (ascending colon) were generated for each experimental group. Whole transcriptome microarray expression analysis (Affymetrix Genechip Mouse Exon 1.0 ST Array®) and bioinformatic analysis was performed. The entire genome-wide expression dataset was used for subsequent analysis [9,50].

### The *in-silico* prioritization of QTL candidate genes

The use of workflows in the analysis of large-scale genomic data provides a systematic and un-biased mechanism for hypothesis generation [51]. Previously constructed workflows were re-used for the analysis of QTL and gene expression data, to identify biological pathways which correlated with *Trichuris muris* infection. The identification of candidate genes underlying each QTL was carried out by firstly determining the precise co-ordinates of each genetic marker (Mbp) (Table 1). Each QTL was subsequently entered into the workflow *qtl_to_pathway* (Additional file 1: Figure S2 [16]). Genes located within each QTL were annotated with additional accession number identifiers (including UniProt ID and Entrez Gene IDs), in order to cross-reference Ensembl database identifiers to KEGG (Kyoto Encyclopaedia of Genes and Genomes) [52] pathway identifiers. As a result, annotated biological pathways were extracted from the KEGG database for inclusion in further analysis.

In parallel, differentially expressed genes identified from the *T. muris* microarray study [9] were analysed using the *refseq_ids_to_pathways* workflow (Additional file 1: Figure S3 [17]). This workflow required preliminary analysis of the gene expression data [9] (Partek Genomic Solution version 6.5, 2009, Partek, USA) and conversion of Affymetrix probe-set identification markers to their recognised NCBI *RefSeq* identification code (*refseq ids*). An identical process to that of the *qtl_to_pathway* workflow for gene annotation was then carried out.

The mapping of gene expression data to KEGG highlighted biological pathway activity in the pathogenesis of colonic disease. All genes with significant transcriptional differences between resistant and susceptible strains, in naïve and infected states (ANOVA, factor interaction, p <0.05), were included for analysis. To identify *cis*-QTL genes of biological relevance to phenotype, those genes with a higher degree of over/under expression (Fold Change ≥ +/-1.4 over naïve levels) during chronic *T. muris* intestinal inflammation, were used in the workflow analysis (see Figure 3).

The workflow *common_pathways* (Additional file 1: Figure S4 [53]) was used to identify candidate pathways containing differentially expressed genes within a QTL, in order to obtain an overall view of the mechanisms which may be influencing the expression of the phenotype.

Additional text mining was used to prevent potential candidate genes which lacked KEGG pathway annotation from being discarded. Transcribed QTL genes were analysed using a text mining workflow (Additional file 1: Figure S5 [54]). Briefly, published abstracts were identified from a PubMed search using the term “(“Colitis” AND “Inflammation”) AND (“Human” OR “Mouse”)”. All scientifically relevant keywords contained within individual abstracts were extracted, constructing a phenotype concept profile and allowing the calculation of inverse document frequency (IDF) scores ie a score relating the number of resulting documents which contained the keywords in question. In parallel, abstracts pertaining to selected genes were similarly recorded. The identification of phenotype keywords within individual gene abstracts allowed for the generation of a cosine vector score for each gene ranging from +1 to -1 (+1 = causation of phenotype; 0 = unknown association with phenotype; -1 = preventative of phenotype). Ranked by their cosine vector score, the association with phenotype of a particular gene was displayed. Similarly, individual phenotype keywords were also ranked according to the IDF scores, identifying possible correlations between each gene and the phenotype. All data regarding text mining and workflow approaches are published online [55].

Only QTL genes known to possess SNP variation between parental AKR and BALB/c [56] were subject to further analysis.

### Independent replication of candidate gene expression by qPCR (*Tm3*)

Infected parental strains AKR and BALB/c (Harlan Olac, UK) received 300 *T. muris* ova by oral gavage. Mice were culled days 0 (naive), 7, 14, 21 and 35 post-infection for analysis (n = 3 for each cohort). mRNA was extracted from 0.5 cm of whole colonic tissue segments, from the ascending colon, according to manufacturer’s instruction (TRIZOL®, Invitrogen). cDNA was synthesised. A full list of gene primers (Eurofins-MWG-Operon, Germany) and their sequences are provided (Additional file 1: Table S1). Samples were quantitatively analysed using KAPA SYBR FAST qPCR Master Mix (Kapa Biosystems Inc., USA) and a Bio-Rad MyIQ™ PCR detection system (Bio-Rad IQ5 optical system software, version 2; Bio-Rad Laboratories Inc.,©). Three replicate cDNA samples were run at a 1:20, a 1:100, and a 1:500 dilutions for each time-point. Threshold cycles were calculated; gene detection within the three serially diluted samples was standardized, and then normalized against housekeeping gene beta-actin (*Act-b*). Relative fold change in gene quantity was calculated using naïve resistant mice as a reference.

## Competing interests

The author declares that they have no competing interests.

## Authors’ contributions

SL: Study design; data acquisition; data analysis; data interpretation drafting and writing of manuscript; obtaining funding. PF: Data analysis concept and design; data acquisition; data analysis; data interpretation; writing of manuscript. JH: Data acquisition; technical support. LZ: Data analysis; bioinformatics. SE: Data analysis; data interpretation. WO: Data analysis; data interpretation. JM: Revision of manuscript. AB: Data analysis concept and design; data interpretation. RG: Study concept and design; obtaining funding; study supervision. JP: Study concept and design; data acquisition; data analysis; data interpretation; drafting and critical revision of manuscript; study supervision. All authors read and approved the final manuscript.

## Authors’ information

Richard K Grencis and Joanne L Pennock co-senior author.

## Supplementary Material

Additional file 1 **Contains Supplementary Figure S1 showing gender variation in phenotype.** Also Supplementary Figures S2-S5 which detail stepwise representations of workflows mentioned in Methods (see legends below), and Table S1 showing primer sequences for qPCR. **Table S1:** Primer sequences for qPCR-amplified genes. **Figure S1:** Phenotype data for whole cohort, stratified by gender. A: Females showed significantly lower worm burden compared to males (Mann Whitney U test, p < 0.0001). B: Females showed significantly higher IgG1:2a antibody ratio (T test, p < 0.0001). **Figure S2:** Pathways and Gene annotations for QTL region. This workflow searches for genes which reside in a QTL (Quantitative Trait Loci) region in the mouse, *Mus musculus*. The workflow requires an input of: a chromosome name or number; a QTL start base pair position; QTL end base pair position. Data is then extracted from BioMart to annotate each of the genes found in this region. The Entrez and UniProt identifiers are then sent to KEGG to obtain KEGG gene identifiers. The KEGG gene identifiers are then used to search for pathways in the KEGG pathway database. (http://www.myexperiment.org/workflows/1661.html). **Figure S3:** Pathways and Gene annotations for RefSeq ids. This workflow searches for *Mus musculus* genes found to be differentially expressed in a microarray study. The workflow requires an input of gene ref_seq identifiers. Data is then extracted from BioMart to annotate each of the genes found for each ref_seq id. The Entrez and UniProt identifiers are then sent to KEGG to obtain KEGG gene identifiers. The KEGG gene identifiers are then used to search for pathways in the KEGG pathway database. (http://www.myexperiment.org/workflows/1662.html). **Figure S4:** KEGG pathways common to both QTL and microarray based investigations. This workflow takes in two lists of KEGG pathway ids. These are designed to come from pathways found from genes in a QTL (Quantitative Trait Loci) region, and from pathways found from genes differentially expressed in a microarray study. By identifying the intersecting pathways from both studies, a more informative picture is obtained of the candidate processes involved in the expression of a phenotype. (http://www.myexperiment.org/workflows/1663.html). **Figure S5:** Pathway and Gene to Pubmed. This workflow takes in a list of gene names, KEGG pathway descriptions and phenotypes as keywords, and searches the PubMed database for corresponding articles. Retrieved abstracts are then used to calculate a cosine vector space between two sets of corpora (gene and phenotype, or pathway and phenotype). The workflow counts the number of articles in the PubMed database in which each term occurs, and identifies the total number of articles in the entire PubMed database so that a term enrichment score may be calculated. Scientiifc terms are then extracted from the abstract text and given a weighting according to the number of terms that appear in the document. The higher the value the better the score. This is given as: X (or Y) = log((a / b) / (c / d)) where: a = number of occurrences of individual terms in phenotype (or pathway) corpus, b = number of abstracts in entire phenotype (or pathway) corpus, c = number of occurrences of individual terms in entire PubMed, d = number of articles in entire PubMed. Once this has been created, the pathways obtained from the QTL and microarray pathway analysis workflows are analysed. The weighted terms are then given a link score X + Y. The higher the score the more “appropriate/interesting” the link between the pathway and the phenotype. This is calculated as: W= (X + Y). (http://www.myexperiment.org/workflows/1846.html). Click here for file
