# Supplementary material for: Genetic analysis of the Trichuris muris-induced model of colitis reveals QTL overlap and a novel gene cluster for establishing colonic inflammation
Source: BMC Genomics. 2013 Feb 26;14:127. doi: 10.1186/1471-2164-14-127 (PMC3621453; doi:10.1186/1471-2164-14-127)
Supplement: Additional file 1 — Contains Supplementary Figure S1 showing gender variation in phenotype. Also Supplementary Figures S2-S5 which detail stepwise representations of workflows mentioned in Methods (see legends below), and Table S1 showing primer sequences for qPCR. Table S1: Primer sequences for qPCR-amplified genes. Figure S1: Phenotype data for whole cohort, stratified by gender. A: Females showed significantly lower worm burden compared to males (Mann Whitney U test, p < 0.0001). B: Females showed significantly higher IgG1:2a antibody ratio (T test, p < 0.0001). Figure S2: Pathways and Gene annotations for QTL region. This workflow searches for genes which reside in a QTL (Quantitative Trait Loci) region in the mouse, Mus musculus. The workflow requires an input of: a chromosome name or number; a QTL start base pair position; QTL end base pair position. Data is then extracted from BioMart to annotate each of the genes found in this region. The Entrez and UniProt identifiers are then sent to KEGG to obtain KEGG gene identifiers. The KEGG gene identifiers are then used to search for pathways in the KEGG pathway database. (http://www.myexperiment.org/workflows/1661.html). Figure S3: Pathways and Gene annotations for RefSeq ids. This workflow searches for Mus musculus genes found to be differentially expressed in a microarray study. The workflow requires an input of gene ref_seq identifiers. Data is then extracted from BioMart to annotate each of the genes found for each ref_seq id. The Entrez and UniProt identifiers are then sent to KEGG to obtain KEGG gene identifiers. The KEGG gene identifiers are then used to search for pathways in the KEGG pathway database. (http://www.myexperiment.org/workflows/1662.html). Figure S4: KEGG pathways common to both QTL and microarray based investigations. This workflow takes in two lists of KEGG pathway ids. These are designed to come from pathways found from genes in a QTL (Quantitative Trait Loci) region, and from pathways found from genes differen [file 1471-2164-14-127-S1.pdf]

Table S1. Primer sequences for qPCR-amplified genes.

Actb	GGCTGTATTCCCCTCCATCG	CAGTTGGTAACAATGCCATGT
Vav3	GCCACACTCCATCAATCTG	GGTCAAATGCCTCAAAAAGTTC
FcgR1	AGAAGCGATGGCGTGTATGA	TTCAGGCTGCTCGGGTCCAC
Ptpn22_distal	AGCCGTGCAAACTTCTTCTACT	CTGAACACGTTCTGTAGGCTTG
Vcam1	GCCTCAACGGTACTTTGGATAC	TCTCCATGAGAACAATGGTGAC
Hmgcs2	ATGATCTTCCACACACCCTTTT	GCCTTCAAAAGAGCCTTGTCTA
Slc22a15	CTCTGCCCTTCATCGTGT	TCACTGCTGACCTTGTCC
S100a10	GCAAACCTGTGAAACGTA	CAGCTCTTCCAAGGACTG
Ctss	TAAAGGGCCTGTCTCTGT	TGCCATCCGAATGTATCC

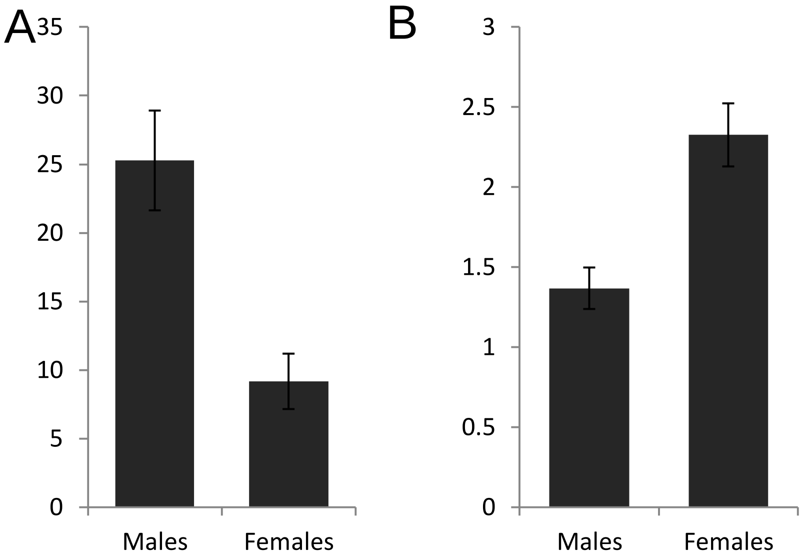

Figure S1

Additional File 1: Levison *et al.* 2012

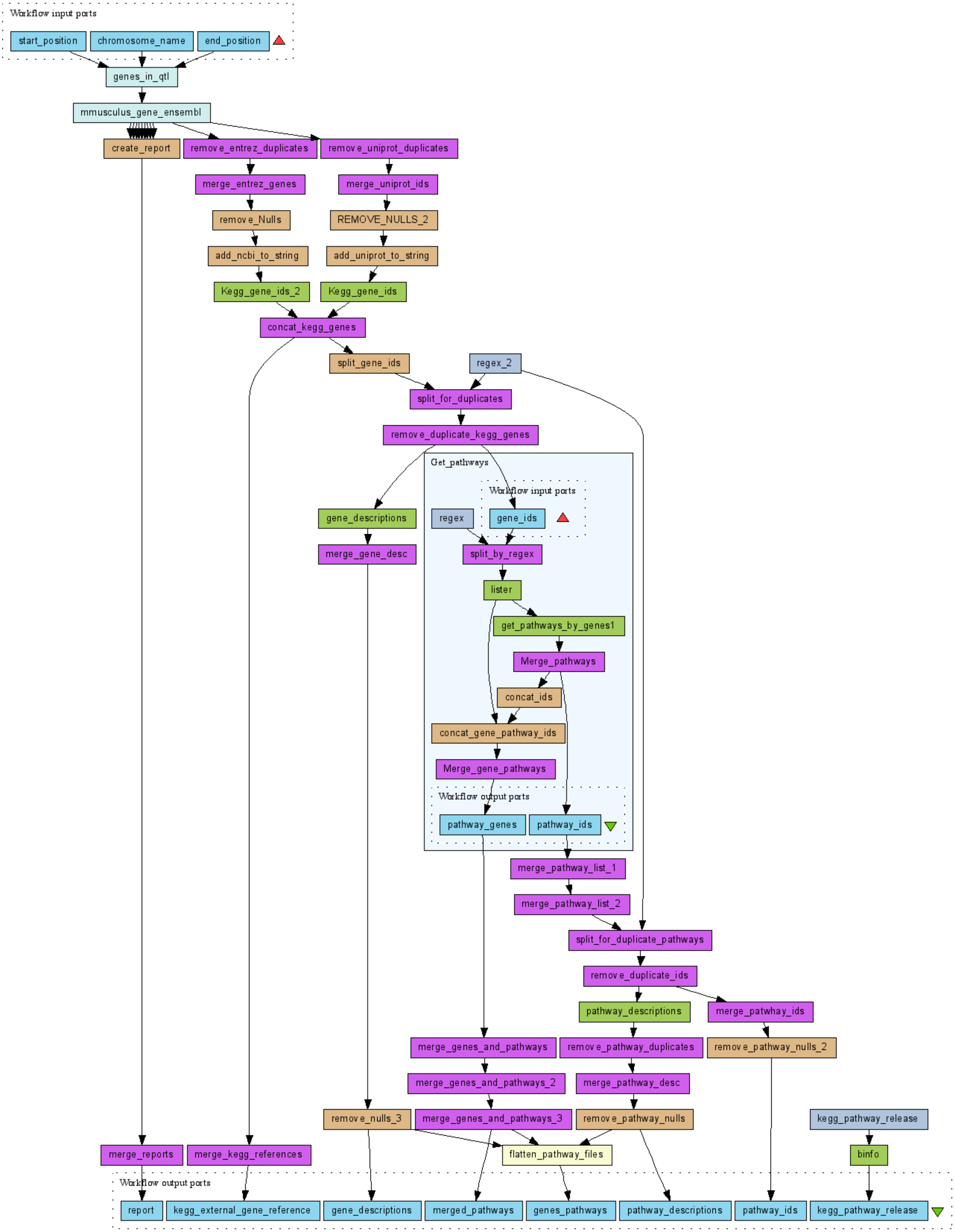

Figure S2

Additional File 1: Levison *et al.* 2012

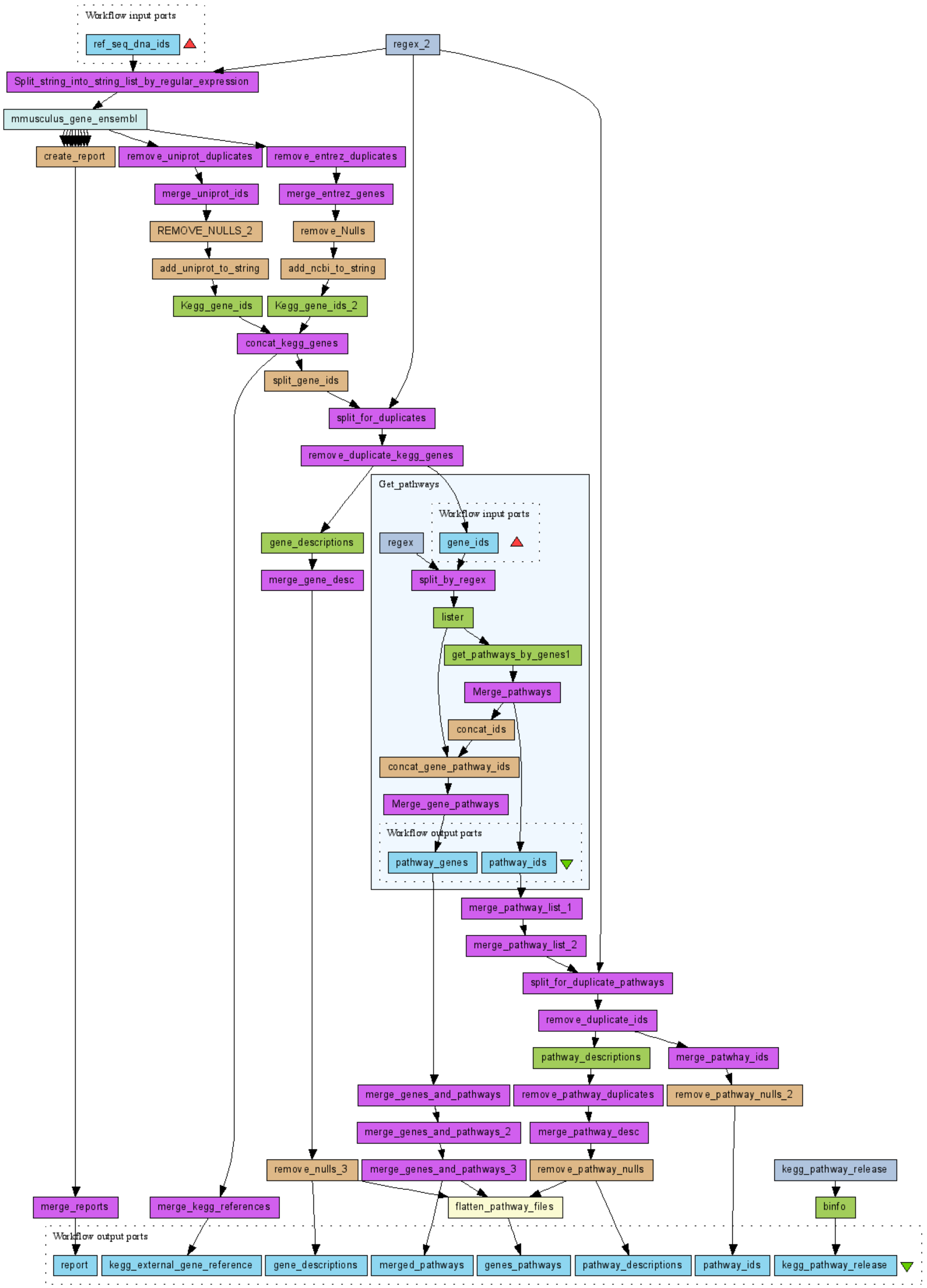
Workflow input ports
ref_seq_dna_ids
regex_2
Split_string_into_string_list_by_regular_expression
mmusculus_gene_ensembl
create_report
remove_uniprot_duplicates
remove_entrez_duplicates
merge_uniprot_ids
merge_entrez_genes
REMOVE_NULLS_2
remove_Nulls
add_uniprot_to_string
add_ncbi_to_string
Kegg_gene_ids
Kegg_gene_ids_2
concat_kegg_genes
split_gene_ids
split_for_duplicates
remove_duplicate_kegg_genes
Get_pathways
Workflow input ports
regex
gene_ids
split_by_regex
lister
get_pathways_by_genes1
Merge_pathways
concat_ids
concat_gene_pathway_ids
Merge_gene_pathways
Workflow output ports
pathway_genes
pathway_ids
gene_descriptions
merge_gene_desc
merge_pathway_list_1
merge_pathway_list_2
split_for_duplicate_pathways
remove_duplicate_ids
pathway_descriptions
merge_patwhay_ids
merge_genes_and_pathways
remove_pathway_duplicates
remove_pathway_nulls_2
merge_genes_and_pathways_2
merge_pathway_desc
remove_nulls_3
merge_genes_and_pathways_3
remove_pathway_nulls
kegg_pathway_release
merge_reports
merge_kegg_references
flatten_pathway_files
binfo
Workflow output ports
report
kegg_external_gene_reference
gene_descriptions
merged_pathways
genes_pathways
pathway_descriptions
pathway_ids
kegg_pathway_release

Figure S3

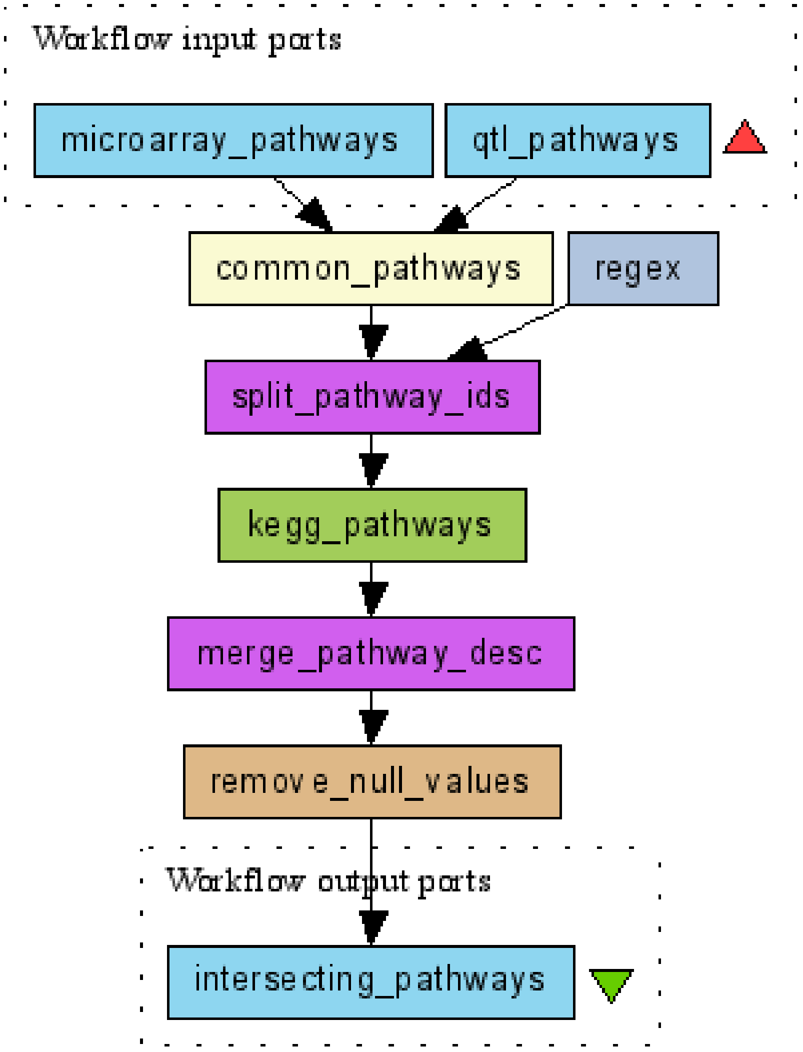
Workflow input ports
microarray_pathways
qtl_pathways
common_pathways
regex
split_pathway_ids
kegg_pathways
merge_pathway_desc
remove_null_values
Workflow output ports
intersecting_pathways

Figure S4

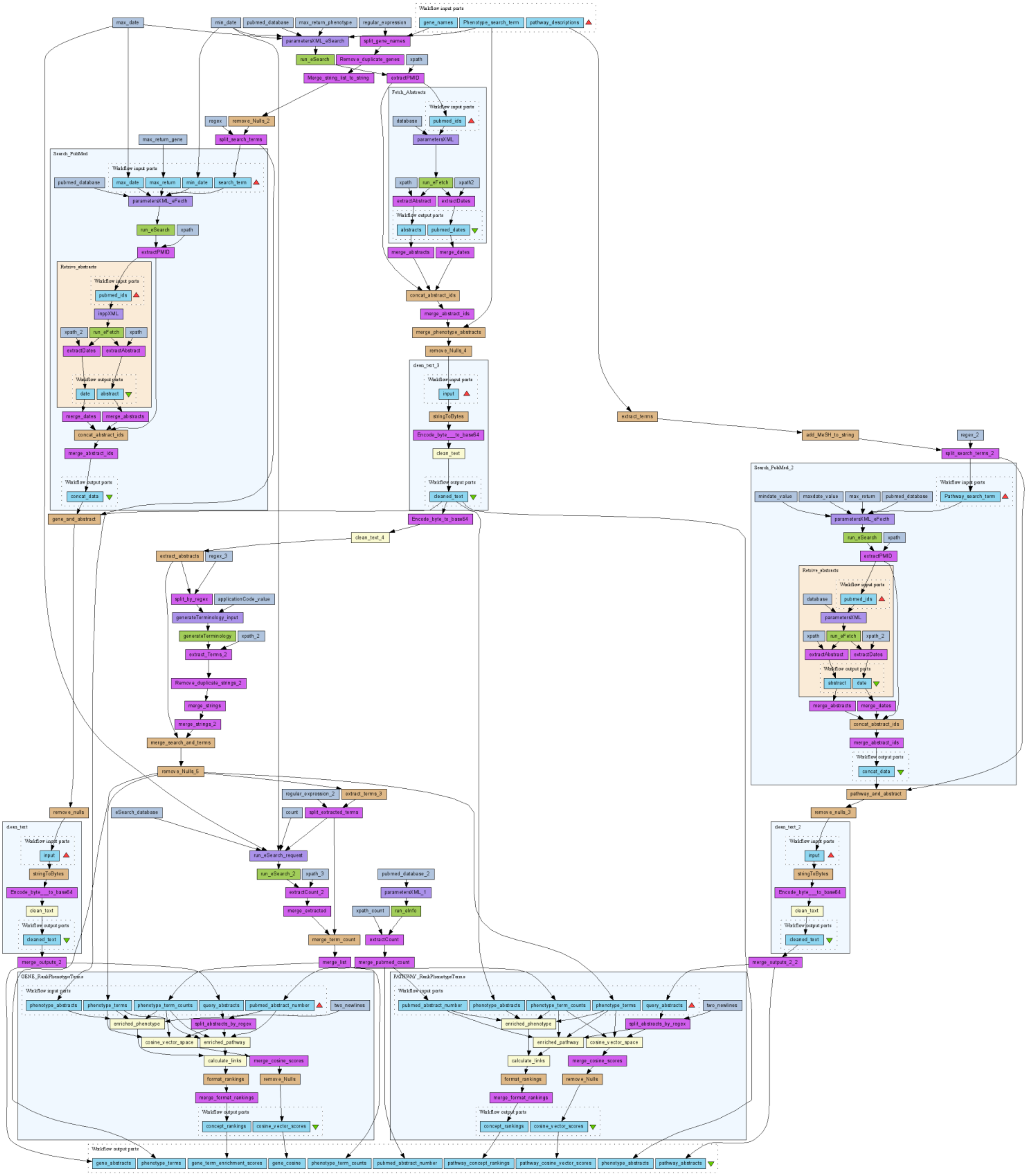

Figure S5

Additional File 1: Levison *et al.* 2012
